# Repurposing Fostamatinib to Combat SARS-CoV-2-Induced Acute Lung Injury

**DOI:** 10.1016/j.xcrm.2020.100145

**Published:** 2020-11-17

**Authors:** Neha Tabassum, Hua Zhang, Justin Stebbing

**Affiliations:** 1 Department of Surgery and Cancer, Imperial College, London W12 0NN, UK; 2 Laura and Isaac Perlmutter Cancer Center, New York University Langone Medical Center, New York, NY 10016, USA

## Abstract

A screen by Kost-Alimova et al.^1^ suggests that the FDA-approved SYK inhibitor fostamatinib inhibits MUC1 in the respiratory tract and has the potential to treat serious outcomes of coronavirus COVID-19, including acute respiratory distress syndrome (ARDS) and acute lung injury (ALI).

## Main Text

The most rapid method to find safe and effective drugs to address COVID-19 is through the repurposing of known drugs for potential use.^2^ Repurposing of well-studied medicines has a number of advantages during the global pandemic, as the toxicity of the drug for *de novo* use has been established for the original indication; this has the potential to shorten development timelines. Although many new therapeutic strategies are being trialed, current treatments for patients infected with severe acute respiratory syndrome coronavirus-2 (SARS-CoV-2) are usually supportive, including immuno-modulatory or anti-inflammatory steroids^3^, or only administered based on emergency use, e.g., remdesivir, originally developed to treat Ebola.^4^ Barictinib, approved for the treatment of rheumatoid arthritis and shows positive activity in the ACTT-II trial, appears to have both anti-viral as well as anti-cytokine activity.^5^ Patients who contract SARS-CoV-2 often develop life-threatening complications, including a severe form of acute lung injury (ALI) known as acute respiratory distress syndrome (ARDS). However, there are limited treatment options for ARDS^6^; thus, identifying a safe and effective treatment for ALI is needed.

Patients with ARDS have elevated levels of Mucin-1 (MUC1), a transmembrane protein expressed on the apical membrane of mucosal epithelial cells, with mucin proteins themselves the major component of mucus. Excessive production of mucus in the airways is linked to a number of different complications including increased duration and frequency of infections, decreased lung function, and increased mortality in patients with respiratory disease.^7^ Previous studies suggest MUC1 might be used as a prognostic marker of SARS-CoV-2 severity, as increased levels of MUC1 in serum have been shown in patients with interstitial pneumonitis and idiopathic pulmonary fibrosis.^8^ In this current study, Kost-Alimova et al.^1^ performed a high-content immunofluorescence imaging screen using MUC1 expression as readout and identified fostamatinib, a SYK inhibitor, as a potent compound that significantly reduced MUC1 protein abundance. Their findings indicated the potential of repurposing fostamatinib (R788) for the treatment of ALI. Indeed, SYK has been used as a therapeutic target in many different roles, especially within the immune system, ranging from arthritis to asthma^9^^,^^10^, and MUC1 has been a well-known albeit failed target in oncology. Fostamatinib was previously approved by the FDA for chronic immune thrombocytopenia.

It is worth noting that the research team had previously focused on identifying MUC1 reducing drugs through the Broad Repurposing Library, targeting a mutant-MUC1 neoprotein causing autosomal dominant tubule-interstitial kidney disease. During the COVID-19 pandemic, the aim shifted to looking for drugs reducing expression of MUC1-wild type (wt) protein to treat SAR-CoV-2-induced ALI. Expression patterns of MUC1 were analyzed using the Human Protein Atlas and MUC1 expression was demonstrated in alveolar epithelium, using immuno-peroxidase staining of human lung tissue, implicating MUC1 in lung function. Imaging from high content screening was quantified using the STAR morphology “Profile” Module, to follow localization of MUC1 on the plasma membrane versus intracellular localization in the presence of test compounds as a readout. Criteria for inclusion of certain drugs over others during screening included the following: reduction of MUC1-wt protein in a dose-dependent manner, favorable toxicity profiles, reduction of MUC1 via non-transcriptional mechanisms, and, finally, they had to be FDA approved.

Over 3,500 drugs from the Library were screened for their ability to reduce MUC1 levels using immortalized kidney tubular epithelial cells. High expression of MUC1 on the plasma membrane of these cells allows for assessment of MUC1 abundance, while cell number is easily determined for the assessment of cell toxicity. Dose response curves were prepared for over 200 positive hits and a further thirteen candidates were then selected for qPCR analysis to determine any correlation between MUC1 protein reduction, MUC1 mRNA reduction, and cell viability screens. One test compound, R406 was shown to reduce plasma membrane MUC1 and increase levels of the protein intracellularly, unlike other compounds tested. Kost-Alimova et al.^1^ only selected test compounds that did not act via transcriptional mechanisms, as there is evidence suggesting low clinical efficacy at the transcriptional level. Therefore, as a result of these criteria, only four drugs were found showing a safe toxicity profile, with only one, R406, already approved by the FDA. Additional SYK inhibitors were also tested, with R406 also having the most potent effect.

The safety profile and tolerability of fostamatinib has been well established in patients with only few presenting with mild to moderate adverse effects that resolved alone or with the help of medical treatment. A mouse model of ALI (ischemia-reperfusion-induced remote lung injury) was used in the current studies to demonstrate that R788, the orally available pro-drug of R406, reduced MUC1 in injured lung epithelia compared to the control, as shown with quantitative imaging analysis. The potential positive effects of repurposing R788 for the treatment of SARS-CoV-2-induced ALI could therefore be promising, although clinical studies are required, which is now problematic as the “sick hospitalization” rate has fallen.

As SARS-CoV-2 is a novel problem needing immediate solutions, animal testing of fostamatinib to determine the effect on SARS-CoV-2-induced ALI has yet to be established. This may be circumvented for fostamatinib, as this is already classified as having a high safety profile through previous FDA approval, has an effect on ALI in an alternate pre-clinical model of ALI and may lead to direct, compassionate use within the target patient population.

In conclusion, this study testing the Broad Repurposing Library allowed for an FDA-approved drug to be discovered with possible benefits to patients presenting with SARS-CoV-2-induced ALI. Although there is no current evidence of fostamatinib being effective in SARS-CoV-2-induced ALI, this drug has been shown to reduce MUC1 in a relevant pre-clinical model and has a demonstrated safety profile in patients.
